# Computational comparison of common event-based differential splicing tools: practical considerations for laboratory researchers

**DOI:** 10.1186/s12859-021-04263-9

**Published:** 2021-06-26

**Authors:** Ittai B. Muller, Stijn Meijers, Peter Kampstra, Steven van Dijk, Michel van Elswijk, Marry Lin, Anna M. Wojtuszkiewicz, Gerrit Jansen, Robert de Jonge, Jacqueline Cloos

**Affiliations:** 1 Department of Clinical Chemistry, Amsterdam UMC – location VUmc, Amsterdam, The Netherlands; 2 ORTEC Netherlands, Zoetermeer, The Netherlands; 3 Department of Hematology, Cancer Center Amsterdam, Rm CCA 4.24, Amsterdam UMC – location VUmc, De Boelelaan 1117, 1081 HV Amsterdam, The Netherlands; 4 Amsterdam Rheumatology and immunology Center, Amsterdam UMC – location VUmc, Amsterdam, The Netherlands

**Keywords:** Alternative splicing, RNA-sequencing, Computational performance

## Abstract

**Background:**

Computational tools analyzing RNA-sequencing data have boosted alternative splicing research by identifying and assessing differentially spliced genes. However, common alternative splicing analysis tools differ substantially in their statistical analyses and general performance. This report compares the computational performance (CPU utilization and RAM usage) of three event-level splicing tools; rMATS, MISO, and SUPPA2. Additionally, concordance between tool outputs was investigated.

**Results:**

Log-linear relations were found between job times and dataset size in all splicing tools and all virtual machine (VM) configurations. MISO had the highest job times for all analyses, irrespective of VM size, while MISO analyses also exceeded maximum CPU utilization on all VM sizes. rMATS and SUPPA2 load averages were relatively low in both size and replicate comparisons, not nearing maximum CPU utilization in the VM simulating the lowest computational power (D2 VM). RAM usage in rMATS and SUPPA2 did not exceed 20% of maximum RAM in both size and replicate comparisons while MISO reached maximum RAM usage in D2 VM analyses for input size. Correlation coefficients of differential splicing analyses showed high correlation (β > 80%) between different tool outputs with the exception of comparisons of retained intron (RI) events between rMATS/MISO and rMATS/SUPPA2 (β < 60%).

**Conclusions:**

Prior to RNA-seq analyses, users should consider job time, amount of replicates and splice event type of interest to determine the optimal alternative splicing tool. In general, rMATS is superior to both MISO and SUPPA2 in computational performance. Analysis outputs show high concordance between tools, with the exception of RI events.

**Supplementary Information:**

The online version contains supplementary material available at 10.1186/s12859-021-04263-9.

## Background

Alternative splicing (AS) is recognized as an important intracellular mechanism of post-transcriptional regulation of gene expression [1, 2]. By splicing of the precursor messenger RNA (pre-mRNA) into a multitude of mRNA variants, the protein-coding potential of eukaryotic genes is expanded, thereby resulting in increased proteome diversity. Over 95% of the human multi-exonic genes are alternatively spliced, and common splice event types include skipped exons (SE), intron retentions (IR), alternative 3’ splice sites (A3SS), alternative 5’ splice sites (A5SS), and mutually exclusive exons (MXE) [3, 4]. Exon skipping occurs when an exon is spliced out together with its flanking introns and this constitutes the most prevalent type of AS events (40% of all AS events). A5SS or A3SS designate events with alternative splice sites that may result in the inclusion or exclusion of alternatively spliced regions, i.e., of alternative parts of exons, while IR are AS events where an entire intron is not spliced out [4].

AS promotes functional diversity of gene regulation, changes in transcript stability or localization as well as the removal or incorporation of post-translational modification sites [5]. As such, AS plays a part in many biological processes involved in normal cellular functions such as homeostasis, differentiation or sex determination [2, 6, 7], but also in disease pathogenesis and pharmacological processes underlying drug resistance [3, 8, 9]. With respect to the latter, AS has been proposed as a source of potential biomarkers or as a target for drug development [10–12]. For example, in childhood acute lymphoblastic leukemia (ALL), an A5SS selection in exon 8 of the folate metabolizing enzyme folylpolyglutamate synthetase (FPGS) was shown to be associated with the clinical response to methotrexate (MTX), an anchor drug in the treatment of ALL [13] and rheumatoid arthritis [14].

With the recent advances in RNA-sequencing (RNA-seq) technologies, whole-transcriptome analysis of AS profiles has become feasible, requiring the development and application of up-to-date/state-of-the-art computational tools to reliably detect common and less common differential splicing (DS) events [6]. DS comprises the analysis of AS in which event or isoform abundance is compared across two or more conditions. Over the past decade, a plethora of different DS tools have been developed and evaluated, many of which harbors the ability to sensitively and accurately detect and quantify AS events [15]. While most tools are similar in execution, it is also important to distinguish tools that analyze isoform-level DS and event-level DS, the latter providing DS information on a specific event type rather than the relative expression of transcript isoforms/variants. Event-level DS tools need to accurately quantify the abundance of reads/transcripts pertaining (as determined by RNA-seq) specifically to the possible AS event types (SE, IR, A5SS, A3SS, MXE). While comparisons have been made between DS tools in general, these comparisons were often on the isoform-level, and also lacked in-depth analysis of unbiased output concordance [16–18].

In addition, comparative studies or tools publications can conceivably include limited comparisons (e.g. using only a set of SE events and not comparing relative splicing abundances), comparisons of outdated versions, and/or a biased selection (e.g. handpicking a set of specific genes with a maximum of two isoforms) [19–21]. For example, selective reporting of just SE events for tool comparisons could raise an erroneous impression that event detection and measured splicing abundance is similar between event types, which may not reflect the complete account. This can result in bias towards tools adept at detecting SE, but not RI/A5SS/A3SS events. Finally, the use of receiver operating curve (ROC) curve analysis to compare DS tools does not inform on the overlap between tool outputs or concordance between quantifications of AS events.

To address these issues, we here aimed to compare three commonly used event-level DS tools (rMATS [19], MISO [20], and SUPPA2 [21]) based on their computational performance as well as to compare concordance between quantification of differential splicing (Percent Spliced In [PSI], or equivalent) of a cell line and its drug-resistant subclone [13].We selected these three tools due to their relative popularity, their use without knowledge of separate programs such as R, and the ability to analyze on the splicing event-level. Furthermore, we compared each tool for its ability to detect a validated AS event involved in drug resistance across various conditions. Finally, we aim to specify the differences in usability of the different DS tools, analyze their (dis)similarities in output as well as provide practical insights for (inexperienced) biomedical researchers intending to investigate DS with one of the available tools.

## Results

### Job times

Three different Microsoft Azure cloud-based virtual machines (VMs) were used to simulate low (D2), medium (D8), and high (D16) computational power (Table 1).

**Table 1:** Microsoft Azure Virtual Machines

Virtual machine name	vCPUs	RAM (GB)	Max IOPS
D2s_v3	2	8	3200
D8s_v3	8	32	12,800
D16s_v3	16	64	25,600

vCPUs: max number of virtual CPUs. RAM (GB): Max RAM. Max IOPS: max input/output operations per second

**Fig. 1:**
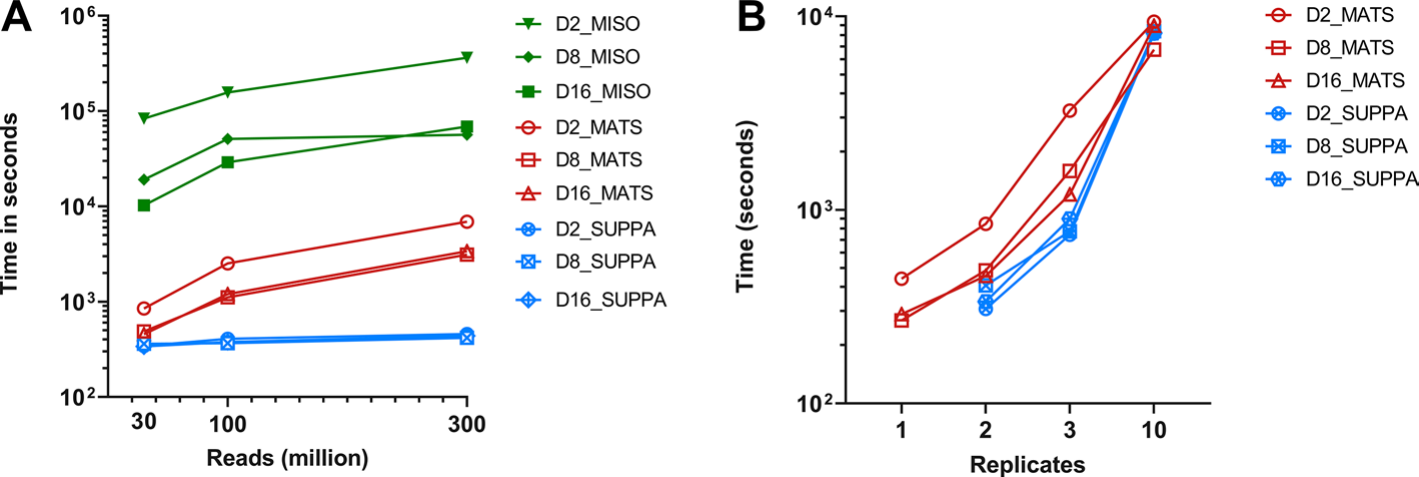
Job times per analysis. Runtime of the different analyses (size (**A**) and replicate (**B**) comparisons) per tool and virtual machine in the simulated dataset. **A** Input size and **B** amount of replicates were varied (x-axis) and time in seconds (y-axis) is shown. Values are in log scale. SUPPA2—blue, rMATS—red, MISO—green

Total job times were calculated for analyzing 2 versus 2 comparisons of different size input files (30 M, 100 M, and 300 M reads) (Fig. 1). Regardless of VM type used, MISO required the longest time to perform the job for each size. Furthermore, both rMATS and MISO showed linear increases in job times when increasing file size in all VMs. Job times for SUPPA2 were consistent regardless of file size, since PSI calculations were performed on transcript expression files, which are identical for each analysis. No difference was seen for rMATS analysis for D8 or D16. In addition, MISO analysis with 300 M reads input did not decrease job time when increasing VM size from D8 to D16.

When analyzing replicates, exponential increases were observed in all rMATS and SUPPA2 analyses. For 10 versus 10 comparisons, D8 rMATS analysis was shown to take the least amount of time at 6719 s (1.9 h). For rMATS, increasing VM size from D8 to D16 increased job times substantially, implying less efficient job performance for D16 than D8 VMs. Job times for SUPPA2 were similar over all analyses regardless of the number of replicates.

### CPU load averages and RAM

**Fig. 2:**
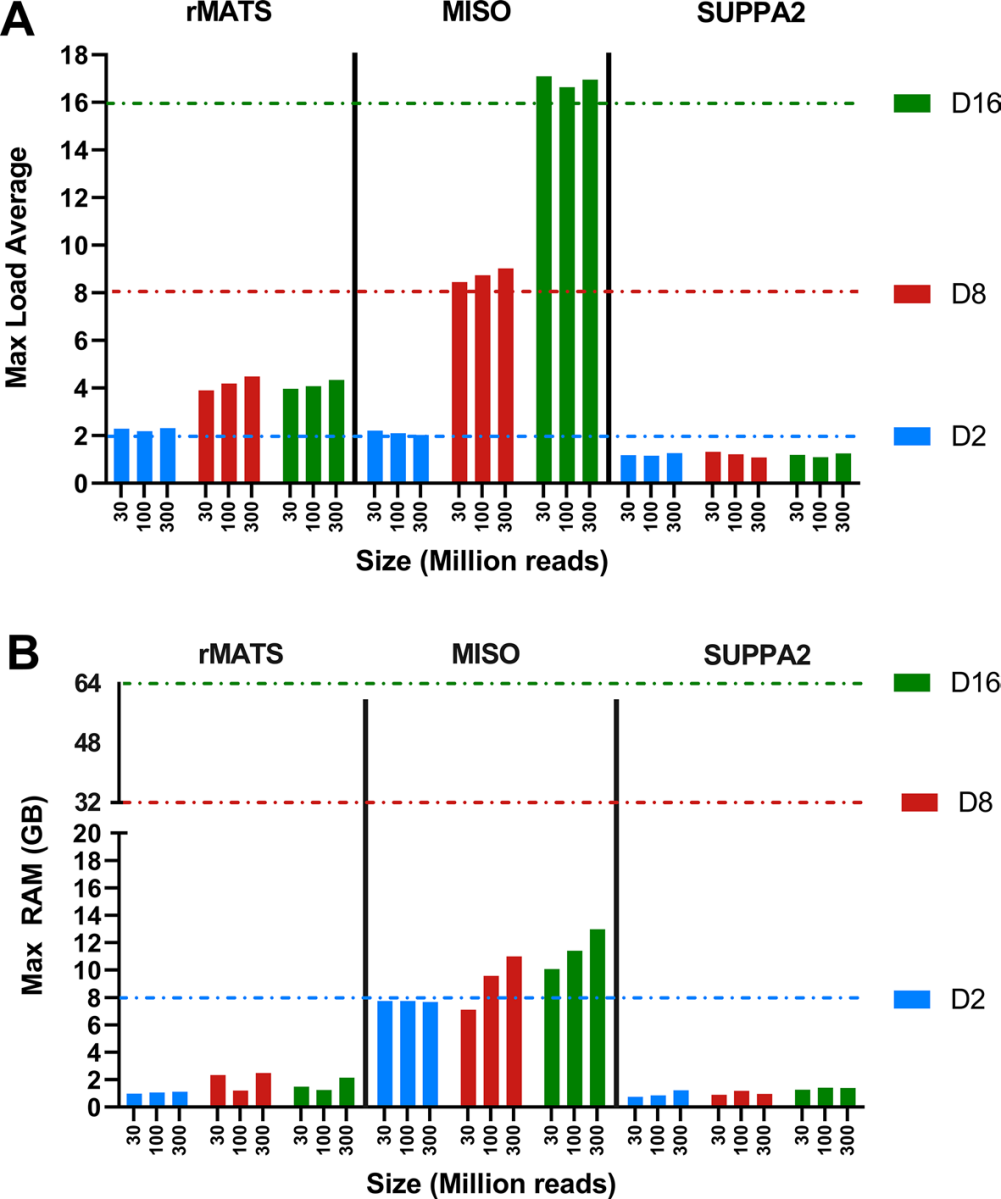
CPU/RAM performance per read depth. **A** Max CPU load averages are plotted for each read depth and VM (max vCPUs: D2–2 (blue dotted line), D8–8 (red dotted line), D16–16 (green dotted line)). **B** Max RAM usage is plotted as percentage of total available RAM. Max RAM per VM: D2–8 GB (blue dotted line), D8–32 GB (red dotted line), D16–64 GB (green dotted line)

**Fig. 3:**
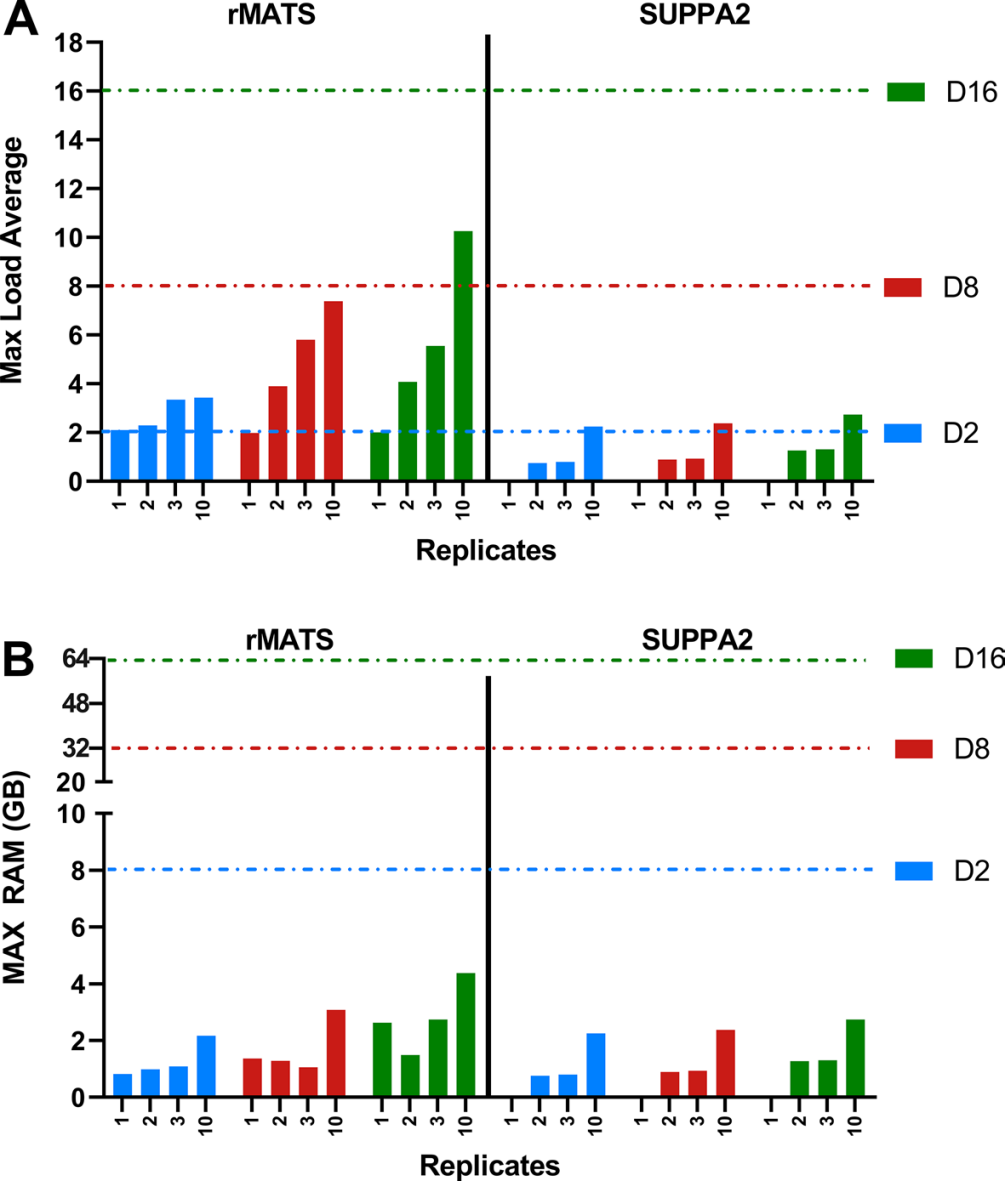
CPU load and RAM usage per replicate. **A** Max CPU load averages are plotted for each amount of replicates and VM (max vCPUs: D2–2 (blue dotted line), D8–8 (red dotted line), D16–16 (green dotted line)). **B** Max RAM usage (in GB) for each replicate and virtual machine. Max RAM per VM: D2–8 GB (blue dotted line), D8–32 GB (red dotted line), D16–64 GB (green dotted line)

VM performance metrics for 2 versus 2 size comparisons are shown in Fig. 2. Maximum load averages of both rMATS and SUPPA2 were relatively low, with a maximum load average of 4.4 for the D16 300 M analysis for rMATS and 1.25 for SUPPA2. In contrast, MISO shows load averages exceeding maximum thread counts for all VM sizes, indicating inefficient CPU utilization. Especially for D2 VM analyses, load averages exceeded CPU numbers substantially, indicating severe CPU thread demand. RAM usage is relatively stable in both rMATS as well as SUPPA2 when using larger VMs, but not when increasing file size (Fig. 2). For MISO, RAM usage in the D2 VM was at maximum (> 95%) for each file size while absolute RAM usage increased with file size for the D8 and D16 VM, not reaching maximum and percentage RAM usage decreased (max 40% and max 20%, respectively (Fig. 2). For replicate analysis, rMATS and SUPPA2 showed increased RAM and CPU load average usage with increased replicate size (Fig. 3), with rMATS using more RAM than SUPPA2 in all analyses. Similar to Fig. 2, rMATS showed limited RAM usage over all analyses, not exceeding 30% of total RAM. Idle RAM usage varied between 1–5%, attributing to non-linear RAM usage in both tools.

### Concordance

**Fig. 4:**
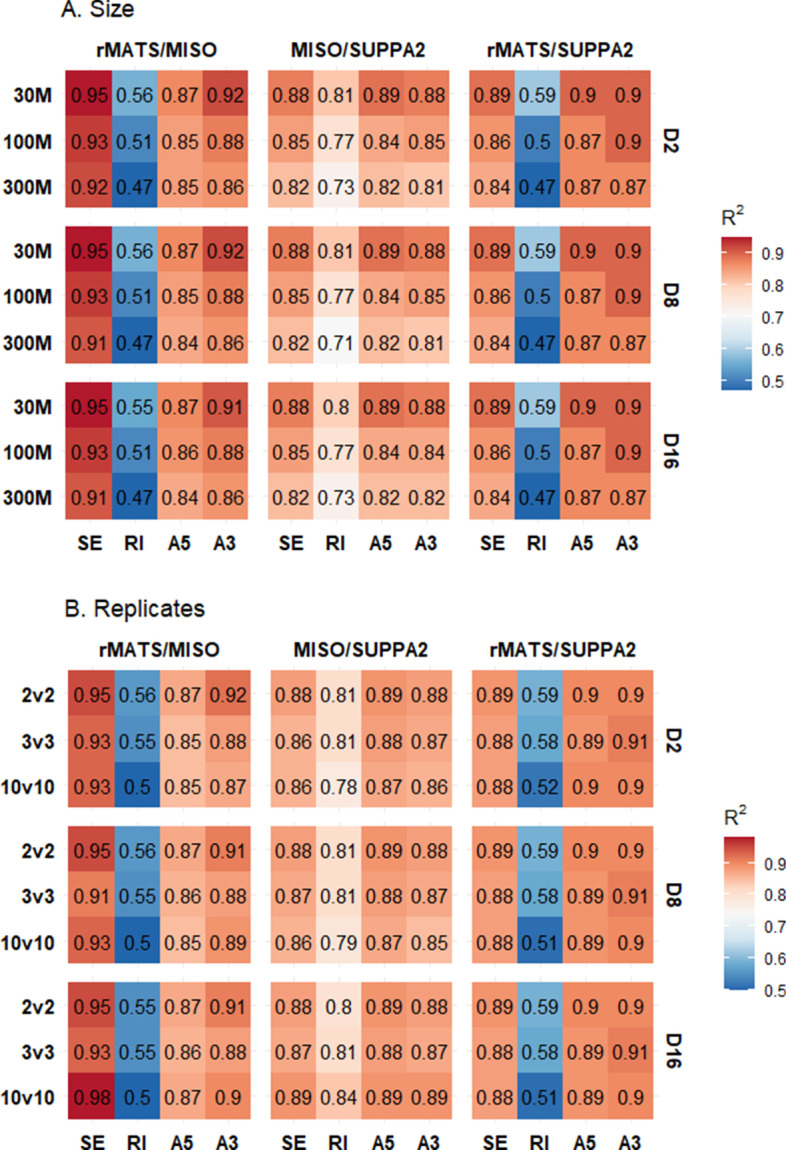
Correlation matrix of concordance of significant matched events between tools. Linear regression coefficients are shown for each read depth (**A**) or amount of replicates (**B**) for each VM per tool combination (rMATS/MISO, MISO/SUPPA2, and rMATS/SUPPA2). Event types: SE–spliced exon, RI–retained intron, A5–alternative 5’splice site, A3–alternative 3’ splice site. D2–D2 virtual machine; D8–D8 virtual machine; D16–D16 virtual machine

**Fig. 5:**
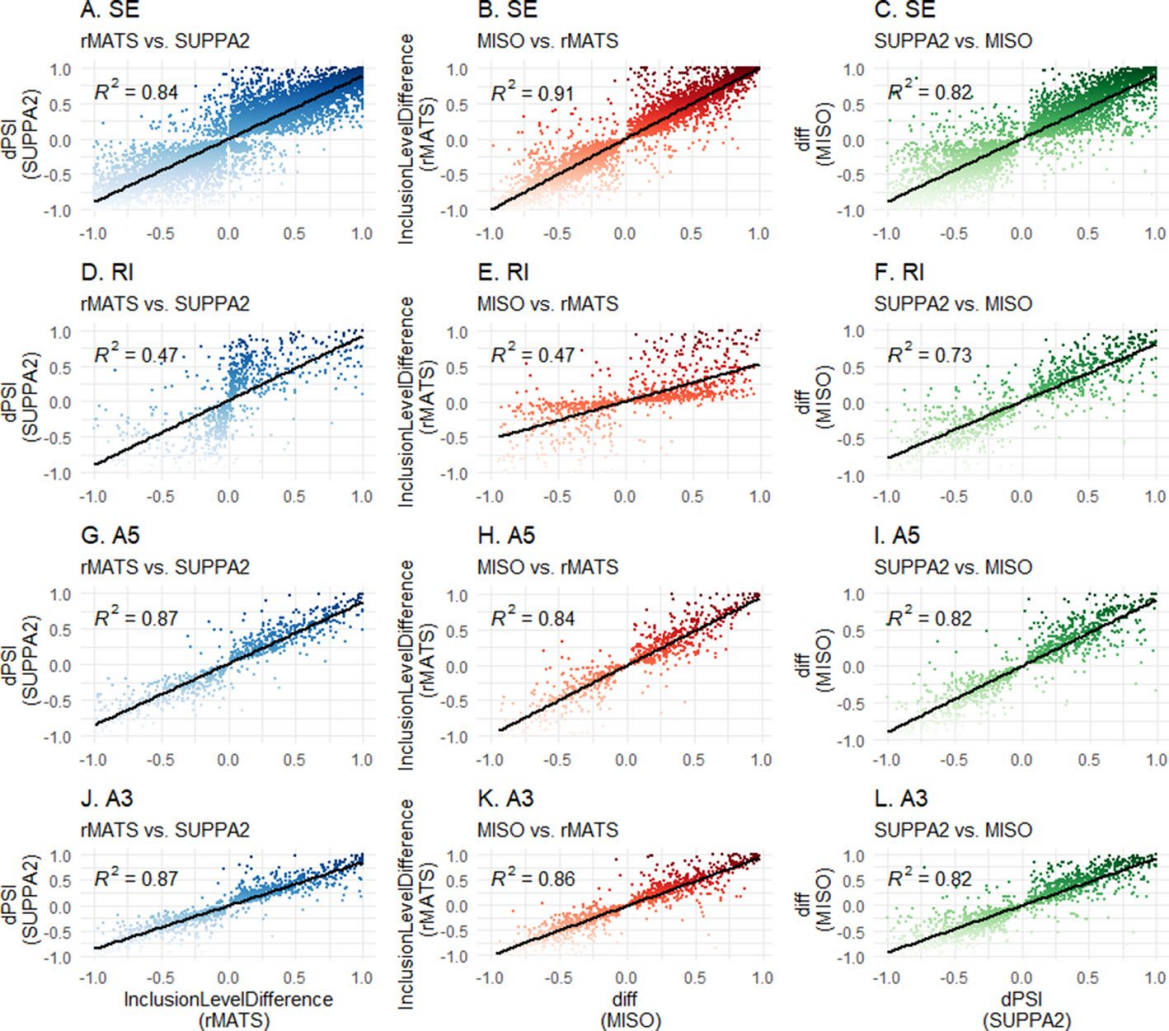
Scatterplots of D16 300 M analyses (significant events) for each event type. Scatterplots show relative splicing abundance for each tool (rMATS–InclusionLevelDifference; MISO–diff; SUPPA2–dPSI) and each event type (SE–spliced exon; RI–retained intron; A5–alternative 5’ splice site; A3–alternative 3’ splice site). Regression coefficient is depicted as R^2^. Linear regression line is shown in black. 5A-C–SE; 5D-F–RI; 5G-I–A5; 5 J-L–A3. Left column–rMATS versus SUPPA2; middle column–MISO versus rMATS; right column–SUPPA2 versus MISO

**Fig. 6:**
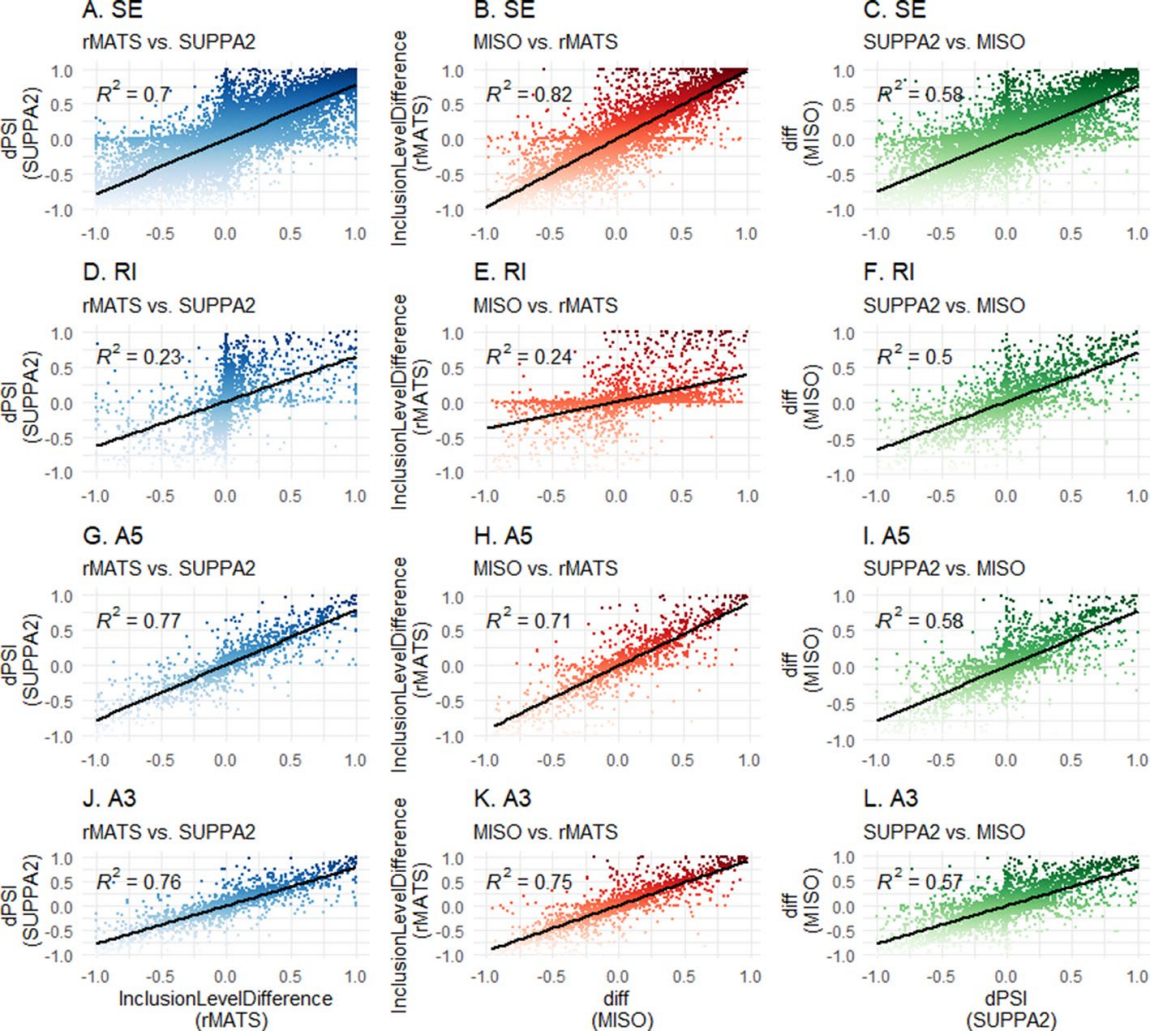
Scatterplots of D16 300 M analyses (all events) for each event type. Scatterplots show relative splicing abundance for each tool (rMATS–InclusionLevelDifference; MISO–diff; SUPPA2–dPSI) and each event type (SE–spliced exon; RI–retained intron; A5–alternative 5’ splice site; A3–alternative 3’ splice site). Regression coefficient is depicted as R^2^. Linear regression line is shown in black. 6A-C–SE events; 6D-F–RI events; 6G-I–A5 events; 6J-L–A3 events. Left column–rMATS versus SUPPA2; middle column–MISO versus rMATS; right column–SUPPA2 versus MISO

To assess concordance, (significant) events of each tool output have been extrapolated, and matched based on their respective coordinates. Correlation coefficients were calculated through linear regression analysis of all (significant) matched splice events. Correlation coefficients shown in Fig. 4 show a general pattern of concordance between tools for SE, A3SS and A5SS events (R^2^ > 0.8 for most comparisons). Notably, rMATS versus MISO comparisons show high similarity in measured PSI for matched SE events (R^2^ > 0.9). However, poor concordance (R^2^ < 0.5) was noted for RI events in the MISO versus rMATS and rMATS versus SUPPA2 comparisons, due to differing relative PSI values for matched RI events, shown in Fig. 5. A similar pattern is seen when including non-significant events (Additonal file 1: Fig. 4). When comparing all events (including non-significant ones) between the splicing tools, correlation coefficients between MISO and rMATS and SUPPA2 and rMATSwere 0.24 and 0.23, respectively (Fig. 6D, E).

No significant differences were observed when comparing outputs between different VM types. However, small differences in significant events were visible when MISO analysis was performed on different VMs, reflected in the slightly different R^2^ values for rMATS versus MISO and MISO versus SUPPA2. This was not observed in the rMATS versus SUPPA2 comparison. Of the RI comparisons, MISO versus SUPPA2 (R^2^ > 0.7) show the best concordance over all sizes or replicates, while rMATS versus MISO and rMATS versus SUPPA2 were the most discordant (R^2^ > 0.4). As a biologically relevant example, a known RT-PCR-validated differentially spliced A5SS event in FPGS (FPGS 8PR), being associated with MTX resistance in CEM/R30dm cells [22], was investigated (Additonal file 1: Fig. 2) and found as significantly differentially spliced in all MISO analyses (Bayes factor > 10) and in the 10 versus 10 rMATS comparisons. The event was found in all other rMATS analyses as well as all SUPPA2 analyses, although statistical significance was not reached (Additonal file 1: Tables 1 and 2).

**Table 2:** Summarizing features of the 3 splicing analyses tools

	rMATS	MISO	SUPPA2
Version	4.0.2	0.5.4	2.3
Release Date	May 2018	July 2017	February 2018
Other required tools	None	Samtools	Kalisto/Salmon
Reference	GTF	GFF3	GTF
Input file	FASTQ/BAM	BAM	TPM
Supported experiments	Two groups, paired	1 versus 1 comparison	Two groups, paired
Job time	++	– –	+
RAM	+ +	– –	+ +
CPU	+ +	– –	+ +
Ease of use	+ +	– –	–
FPGS 8PR detection	+/−	+	+/−

++ , very good; + , good;  +/− , average; –, poor; ––, very poor

## Discussion

In this report, we compared three commonly used event-level AS tools with regard to their computational performance metrics as well as whether the tools produce similar output (Table 2). We aimed to inform wet-lab researchers who want to start working in this field and discuss on the possibilities and pitfalls of splicing analysis of RNA-seq data by applying commonly used tools. Obviously, a plethora of other tools exist that could have been added to the current study. We purposefully did not include R-based tools, since that would require knowledge on a separate programming language to operate.

Our results show that different splicing tools are often concordant, but show different outputs per each type of event and the measured relative splicing abundance can be different between tools. Therefore, presenting SE-only analyses or plots representative of the most abundant and easily detectable type of splicing event does not reveal the complete picture. For example, assessing rMATS requires SE events as well as RI events, since it is observed that relative splicing abundance for RI is underestimated compared to MISO/SUPPA2 (Fig. 5D, E). This information is not revealed in ROC curve analyses, and future comparisons of splicing tools should include investigation of multiple splicing event types as well as explore relative splicing abundance.

Furthermore, our results showed that both rMATS and SUPPA2 were superior to MISO with regards to their performance (job times, CPU load averages, and RAM usage). Although MISO—like rMATS and SUPPA2—applies multithreading to its analyses, high load averages indicate high CPU demand while RAM usage is relatively high.

The use of load average over CPU usage can be argued. We reasoned that information on whether a tool reached maximum CPU usage is less valuable than whether requested CPU threads exceeded CPU availability (which would show as 100% CPU). Load averages exceeding the maximum CPU capacities can therefore not be demonstrated by using CPU usage, such as in a study by Ding et al. [16]. In our analysis, MISO would usually be at 100% CPU usage in D2 VM analyses, which is little informative on actual CPU utilization. One significant caveat in the assessment of performance of the splicing tools relates to the decision to exclude pre-processing jobs in the performance calculations. In particular, SUPPA2 relies on transcript expression calculations performed by a separate tool like Kalisto or Salmon [23, 24].

In addition, a possible risk of using virtual CPUs over physical computers is the lack of control over using the exact same instance of VM for each analysis. Although we did not experience adverse effects on our analysis, it is known that Azure Cloud users can get a VM assigned with different specifications as requested, potentially influencing job times/performance.

Due to the majority of job time and CPU/RAM usage being necessary in these prior calculations, SUPPA2 obtains a strong advantage over rMATS and MISO. However, when comparing 10 versus 10 analyses with 3 versus 3 analyses, the increase in CPU load averages and RAM for SUPPA2 was markedly higher than for rMATS, indicating a poorer efficiency when increasing sample sizes. Indeed, this is confirmed in a study by Mehmood et al*.* [18], where SUPPA2 showed the largest increase in RAM usage when increasing the amount of replicates per analysis.

Overall, we observed a significant increase in performance when using a D8 VM over a D2 VM, while the performance of a D16 did not differ greatly from a D8 VM. Of course, this is highly dependent on the input size of the sample sets and quite likely, larger datasets (50 per group or more) would benefit from larger VM usage. We considered a D8 VM similar to standard office workstations, making most analyses possible without the use of a high-end computer system or expensive cloud solutions. However, it must be noted that some pre-processing tools, such as STAR require large amounts of RAM not present in a D8 VM or lower as well as taking significantly more time than splicing analysis.

The underlying statistical frameworks of each tool are different and filtering on p-value in SUPPA2 is more stringent than filtering on FDR in rMATS (e.g. at D16 300M comparison: 11074 significant events for SUPPA2 versus 14868 for rMATS). In addition, if an FDR cut-off of < 0.01 for rMATS (14069 events) is applied, it would still filter less events than p-value filtering for SUPPA2 and the difference in significant events between p-value and FDR in rMATS is merely 1% (14868 versus 14068 events). Aligning each tool for their statistical cut-off to be equally stringent is challenging since MISO uses a Bayesian framework, which does not use p-values at all. In our analyses, we therefore chose the most stringent filtering at an alpha of 5% for rMATS and SUPPA2 and used the cutoffs recommended in the respective software manuals. While concordance between measured PSI values between the tools was high in SE, A3SS and A5SS event types, rMATS showed poor concordance with both SUPPA2 and MISO in RI event types. Which of the tools is closer to the actual RI splicing abundance in these comparisons is not possible to assess without independent validation.

In addition, an A5SS event in FPGS (FPGS 8PR), associated with MTX resistance in CEM/R30dm cells [22], was observed (Additonal file 1: Fig. 2) as significantly differentially spliced in all MISO analyses (Bayes factor > 10) and in the 10 versus 10 rMATS comparisons (FDR < 0.05). The event was also identified in all other rMATS analyses as well as all SUPPA2 analyses, although statistical significance was not reached (Additonal file 1: Tables 1 and 2). However, more validated splice events should be investigated to attempt to infer superiority of a splicing tool and this result indicates the need for caution when performing analysis with a single splicing tool.

Interestingly, R^2^ values differed slightly when increasing the VM size during MISO analyses (e.g. D8 30 M MISO versus SUPPA2; R^2^ = 0.81 and D16 30 M MISO versus SUPPA2; R^2^ = 0.80) although it is unclear what is the cause.

Finally, we assessed whether a particular tool is easy to work with. rMATS is relatively simple to use in our experience; only a single command was required to perform the entire analysis. Both SUPPA2 and MISO require substantial bioinformatics knowledge during the pre-processing steps and are multi-step, multi-command pipelines that are prone to errors. In particular, setting up a working MISO pipeline can take substantial time and effort and troubleshooting can be difficult.

In contrast, rMATS has a relatively active online troubleshooting community, and creators or users usually respond rapidly [25]. Also, while rMATS and SUPPA2 have been updated regularly in the past years, MISO has not, which is reflected in its poor ease-of-use and its weak applicability to improved computer architecture.

## Conclusions

Comparing three commonly used AS detection/quantification tools (rMATS, MISO and SUPPA2) revealed that rMATS and SUPPA2 are relatively easy to use and require standard computational processing power in contrast to MISO. A concluding overview of the features, strengths and weaknesses of the three tools is presented in Table 2. With the increase of widely available high performance computing resources, we would recommend that researchers interested in performing splicing analysis on RNA-seq datasets combine both rMATS and SUPPA2 to quickly get results that are immediately independently validated with a second tool.

## Methods

### RNA-sequencing

To compare AS tools, we used RNA-seq datasets from two human T-cell acute lymphoblastic leukemia (T-ALL), CCRF-CEM (ATCC, Manassas, VA) and its subclone CEM/R30dm (provided by Dr. J. McGuire [26]). The cell line CEM/R30dm has been made resistant to MTX by repeated exposure to MTX and has been shown to harbor specific splice variants in FPGS, an enzyme critically involved in metabolism and intracellular retention of MTX [27–29]. Loss of FPGS function, e.g. due to AS, is implicated in MTX resistance [13, 30]*.* The choice for this cell line model for the simulation experiments was based on the proven clinical and pathological relevance of the validated AS event implicated in (MTX) therapy resistance [13, 22, 26, 29, 30]. These cell lines provide a practical case for laboratory researchers providing perspective on tool applicability and output variation in a controlled setting. Using this cell line model we were able to construct datasets of different sizes and number of replicates with similar expression levels.

From these two cell lines, RNA was isolated using the RNeasy Mini Kit (Qiagen), and RNA-seq libraries were constructed with TruSeq mRNA Stranded Kit (Illumina). Samples were run in biological duplicates on a Hiseq2500 RNA-seq system using a single-read 100 base pair protocol [9, 31]. Resulting FASTQ files were trimmed using trimmomatic [32], followed by transcript quantification (in transcript per kilobase million (TPM)) with Stringtie [33].

### Flux simulator

Using Flux Simulator [34], expression files in FASTQ format were simulated by using the transcript expression files generated from Stringtie. Using default settings for Flux Simulator, duplicates of both cell lines with different sizes (30, 100 and 300 million reads) were created, and in addition, ten replicates of 30 million reads each were created in order to simulate different differential splicing experiments.

### STAR

Alignment was performed using the default settings of STAR (Version 2.4 [35]) with the exception of –alignEndsType EndToEnd to remove soft-clipping of the reads, -outSAMtype BAM to produce .bam files and –sdjbOverhang 100 for optimal splice junction overhang length.

### rMATS

rMATS (Version 4.0.2 [19]) detects differential splicing by using a hierarchical framework to model exon inclusion levels [denoted as ψ, or PSI], and takes into account estimation uncertainty in individual replicates and variability among replicates. rMATS does this by first calculating the inclusion and exclusion levels of the exons with the read counts of mapped reads. The PSI is defined as the “*percentage of the exon inclusion transcripts that splice from the upstream exon into the alternative exon and then into the downstream exon, among all such exon inclusion transcripts plus exon skipping transcripts that splice from the upstream exon directly into the downstream exon.*” [19]

### MISO

MISO (Version 0.5.4 [20]) calculates the expression levels of alternatively spliced genes from RNA-seq data, and analyses differentially-expressed isoforms or exons across samples. MISO computes the probability that a mapped read originated from a particular isoform using a Bayesian inference framework. MISO has a multithreading option and was given the maximum amount of threads for each analysis, which is utilized completely by dividing the job into batch jobs equivalent to the thread number. MISO can only analyze single datasets and as such, replicate analysis was not possible.

### SUPPA2

SUPPA2 (Version 2.2 [21]) generates events from a .GTF file and looks at every transcript that contributes to each event. For each transcript, SUPPA2 calculates the PSI. Separately, SUPPA2 uses the transcript per million (TPM) values of each transcript to calculate differential splicing per event. An overview of the features of the three tools is provided in Additonal file 1: Fig. 1.

### Performance

To benchmark the AS tools, technical performance indicators were tracked throughout the different pipelines: job time, CPU load average, and RAM usage. During the entire process, load averages (1 min) were extrapolated from Linux top command every 10 s, and maximum load averages were calculated to show whether each VM could provide the requested CPU threads during the process. Load averages indicate the requested CPU utilization within a certain timeframe (1 min, 5 min or 15 min). Using the load averages, we assessed whether a process requires more CPU power than is currently available (and whether jobs are waiting to be processed). For a D2 VM, with 2 virtual CPUs, a load average above 2 means that there are more threads waiting to process than there are available virtual CPUs and this implies inefficient CPU usage (and latency). By examining the tools for exceeding maximum load averages during jobs, we infer inefficient CPU utilization over the entire tool process.

RAM usage was similarly obtained by dividing the used physical memory parameter by the total physical memory parameter in Linux free command. Pipeline components that were part of pre-processing (such as GFF indexing for MISO) or used a different tool (Samtools for MISO or Salmon for SUPPA2) were excluded, resulting in timing the pipelines specific for each tool and essential for each run (Additonal file 1: Fig. 1). All comparisons are executed on Linux virtual machines (VMs, specifically referred to as D2, D8 and D16) using Microsoft Azure Cloud Services (Table 2). For all analyses, maximum number of threads was specified for each tool and VM and load averages exceeding maximum thread counts were considered inefficient.

### Concordance

Splice events were matched (based on event coordinates) between tools and splicing values (rMATS, inclusion level difference; MISO, diff; SUPPA2, PSI) were plotted. Significance (rMATS, FDR < 0.05; MISO, Bayes factor > 10; SUPPA2, p-value < 0.05) was considered based on the tool respective manuals. While rMATS uses multiple testing correction to calculate FDR, SUPPA2 does not perform multiple testing correction. The most stringent statistics was used for each tool. Linear regression was performed for each combination of tools per splice event type (SE, RI, A5SS, and A3SS). MXE were excluded. Regression coefficients were calculated and plotted.

### (RT-)PCR

After RNA isolation (according to manufacturer’s protocol, RNeasy Mini Kit (Qiagen)), cDNA was synthesized using reverse transcriptase Moloney Murine Leukemia Virus (M-MLV; Invitrogen). cDNA synthesis reaction mixture (total volume 40 µl) contained: 1.5 μl M-MLV, 1 μg RNA, 0.8 μl random hexamer primers (Roche), 1.6 μl of 25 mM dNTPs (Roche), and 1 μl RNAse OUT (Invitrogen). For end-point PCR, sample reaction mixture was made by adding 12.5 µl GoTaq G2 Master Mix (Promega), 1 µl forward primer, 1 µl reverse primer (forward primer 5’-CGCCTCTACCACCGGCTGGA-3’ and reverse primer 5’-GCTCGGTCCCTCAGCACTGC-3’), 1 µl of cDNA and 9.5 µl H_2_O to a total of 25 µl. Samples were run in a standard thermocycler using the following program: 5 min at 95°C, then 31 cycles of: 1 min at 95°C, 1 min at annealing temperature, and 1 min at 72°C, followed by 10 min at 72°C and cooling down to 4°C.

To perform RT-PCR, LightCycler® 480 SYBR Green I Master kit (Roche) was used (total reaction volume of 20 μl) including 10 μl Master Mix 2 × concentrated, 0.25 μM forward/reverse primer (Primer sequences were as follows: 8WT Forward: ACTGCACCAACATCATCAGGAA. 8WT Reverse: AGGGACACCTTGCTTAAAGATG. 8PR Forward: ACTGCACCAACATCATCAGGAA. 8PR Reverse: AGTCTGCCTGGTCACCTTAAAGAT.) and 12.5 ng cDNA. RT-PCR was performed using a LightCycler®480 Instrument II (Roche). Relative expression of the genes was calculated using LightCycler® 480 Software (Version 1.5.1.62, Roche) with β-glucuronidase (GUSB) as reference gene.

## Supplementary Information

**Additional file 1**: Overview of supplemental figures and data.

## Data Availability

Supporting data for this manuscript has been submitted to Gene Expression Omnibus (GEO) under GEO Accession number GSE161144.

## Abbreviations

A3SS: Alternative 3′ splice site
A5SS: Alternative 5′ splice site
ALL: Acute lymphoblastic leukemia
AS: Alternative splicing
CPU: Central processing unit
DS: Differential splicing
FPGS: Folylpolyglutamate synthetase
MXE: Mutually exclusive exon
Pre-mRNA: Precursor messenger RNA
PSI: Percent spliced in
RAM: Random-access memory
RI: Retained intron
RNA-seq: RNA-sequencing
ROC: Receiver operating curve
SE: Skipped exon
VM: Virtual machine
