# Supplementary material for: Computational comparison of common event-based differential splicing tools: practical considerations for laboratory researchers
Source: BMC Bioinformatics. 2021 Jun 26;22:347. doi: 10.1186/s12859-021-04263-9 (PMC8236165; doi:10.1186/s12859-021-04263-9)
Supplement: Supplementary file 1 — Additional file 1: Overview of supplemental figures and data. [file 12859_2021_4263_MOESM1_ESM.pdf]

Supplemental Figure 1. Overview of analyses for each splicing tool

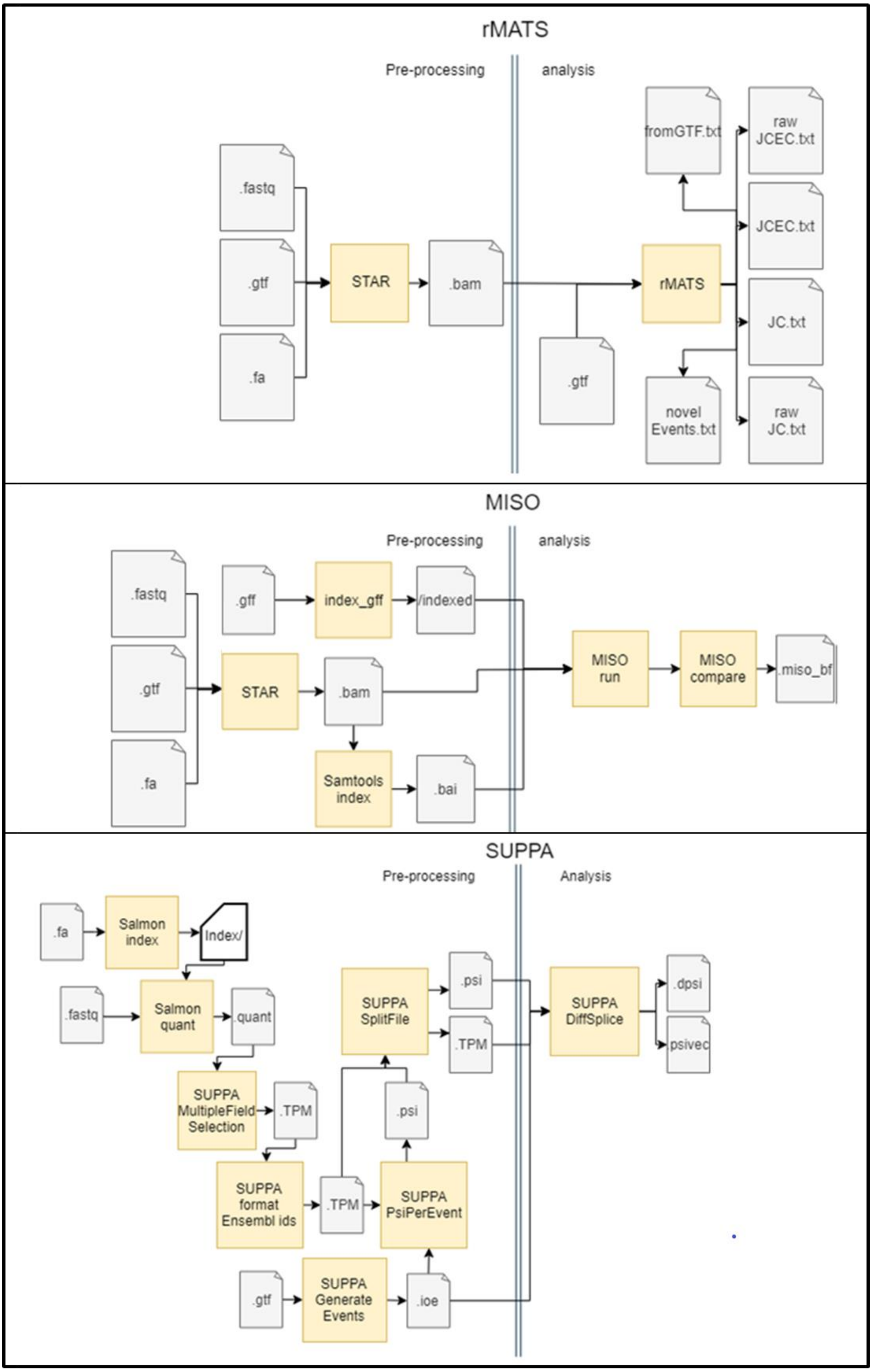

Supplemental Table 1. Overview of FPGS 8PR A5SS splice event calculations for size comparisons.

SIZE			
**rMATS**			
**VM**	**Size**	**Inclusion Level Difference**	**FDR**
D2	30 M	-0.010	0.753
	100 M	0.018	0.363
	300 M	-0.010	0.33
D8	30 M	-0.010	0.753
	100 M	0.018	0.363
	300 M	-0.010	0.33
D16	30 M	-0.010	0.753
	100 M	0.018	0.363
	300 M	-0.010	0.33
**MISO**			
**VM**	**Size**	**Diff**	**Bayes factor**
D2	30 M	0.23	105.50
	100 M	0.19	1.00E+12
	300 M	0.16	1.00E+12
D8	30 M	0.23	163.68
	100 M	0.18	1.00E+12
	300 M	0.16	1.00E+12
D16	30 M	0.23	4.59E+04
	100 M	0.18	1.00E+12
	300 M	0.16	1.00E+12
**SUPPA2**			
**VM**	**Size**	**PSI**	**p-value**
D2	30 M	0.005	0.40
	100 M	-0.003	0.43
	300 M	0.060	0.068
D8	30 M	0.005	0.40
	100 M	-0.003	0.43
	300 M	0.060	0.068
D16	30 M	0.005	0.40
	100 M	-0.003	0.43
	300 M	0.060	0.068

Supplemental Table 2. Overview of FPGS 8PR A5SS splice event calculations for replicate analysis.

REPLICATES			
**rMATS**			
**VM**	**Replicates**	**Inclusion Level Difference**	**FDR**
D2	2 vs. 2	-0.010	0.75
	3 vs. 3	-0.018	0.409
	10 vs. 10	-0.016	0.049
D8	2 vs. 2	-0.010	0.75
	3 vs. 3	-0.018	0.409
	10 vs. 10	-0.016	0.049
D16	2 vs. 2	-0.010	0.75
	3 vs. 3	-0.018	0.409
	10 vs. 10	-0.016	0.049
**MISO**			
**VM**	**Replicates**	**Diff**	**Bayes factor**
D2	2 vs. 2	0.23	105.50
	3 vs. 3	0.24	4.18E+10
	10 vs. 10	0.13	6.93E+03
D8	2 vs. 2	0.23	163.68
	3 vs. 3	0.25	1.00E+12
	10 vs. 10	0.13	6.93E+02
D16	2 vs. 2	0.23	4.59E+04
	3 vs. 3	0.25	9.50E+07
	10 vs. 10	0.35	4.36E+05
**SUPPA2**			
**SVM**	**Replicates**	**PSI**	**p-value**
D2	2 vs. 2	0.005	0.40
	3 vs. 3	0.020	0.29
	10 vs. 10	0.057	0.24
D8	2 vs. 2	0.005	0.40
	3 vs. 3	0.020	0.29
	10 vs. 10	0.057	0.24
D16	2 vs. 2	0.005	0.40
	3 vs. 3	0.020	0.29
	10 vs. 10	0.057	0.24

Supplemental Figure 2

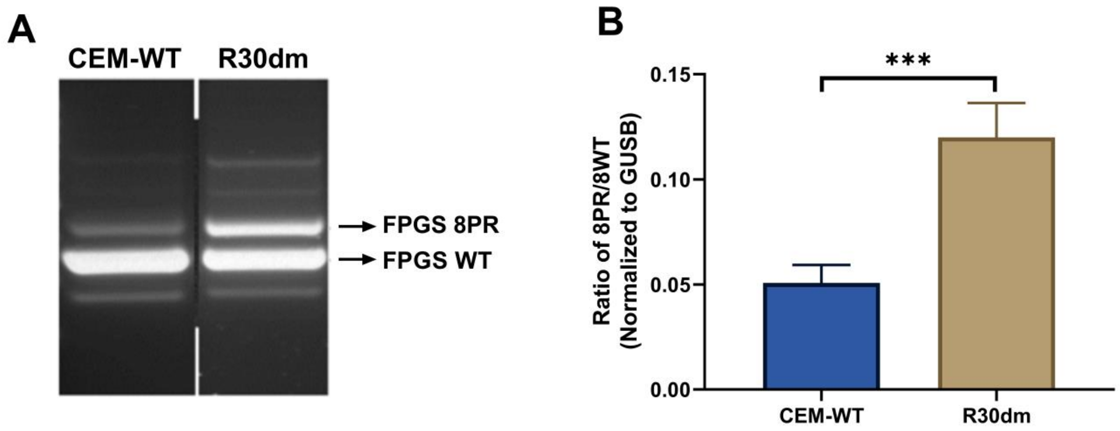

Supplemental Figure 2. (RT)-PCR of FPGS 8PR and FPGS WT.

A) Cropped blot of end-point PCR for FPGS exon 5 to exon 9 in CEM-WT and R30dm cell lines. Upper band indicates partial retention of exon 8 of FPGS. Lower band indicates wild-type variant of FPGS. B) RT-PCR for FPGS 8PR and FPGS 8WT. Relative quantification was performed and was normalized using GUSB. Ratio of FPGS 8PR over FPGS 8WT were calculated and plotted. CEM-WT and CEM-R30dm were significantly different (Student's t-test, p=0.003).

Supplemental Figure 3

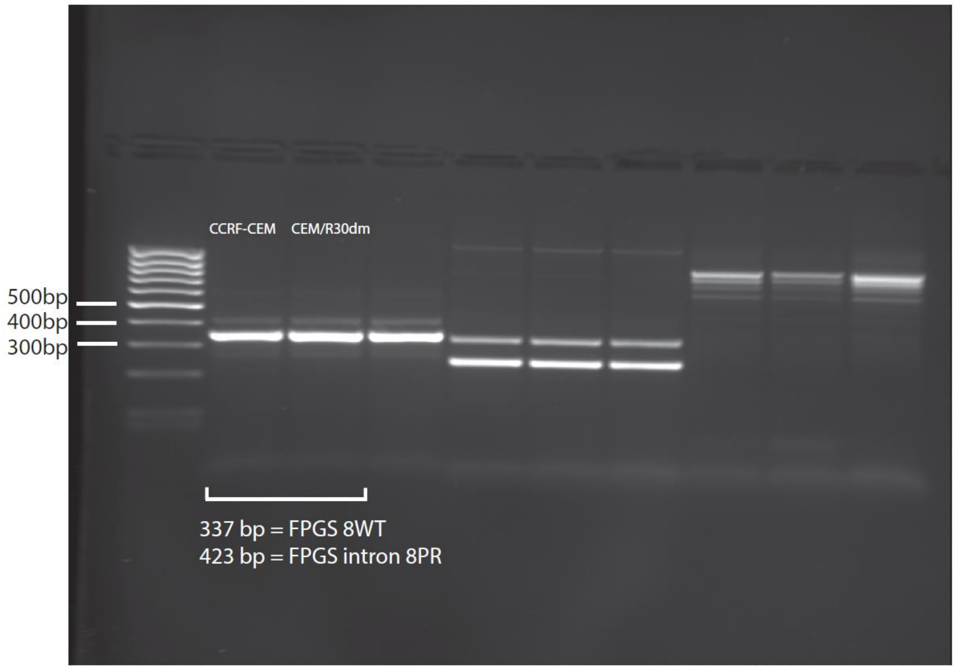

PCR Gel Electrophoresis of CCRF-CEM and CEM/R30dm

Uncropped blot of Figure 5 with expression of FPGS 8 wild-type (FPGS 8WT) and FPGS partial retention intron 8 (FPGS 8PR). bp = base pairs

Supplemental Figure 4.

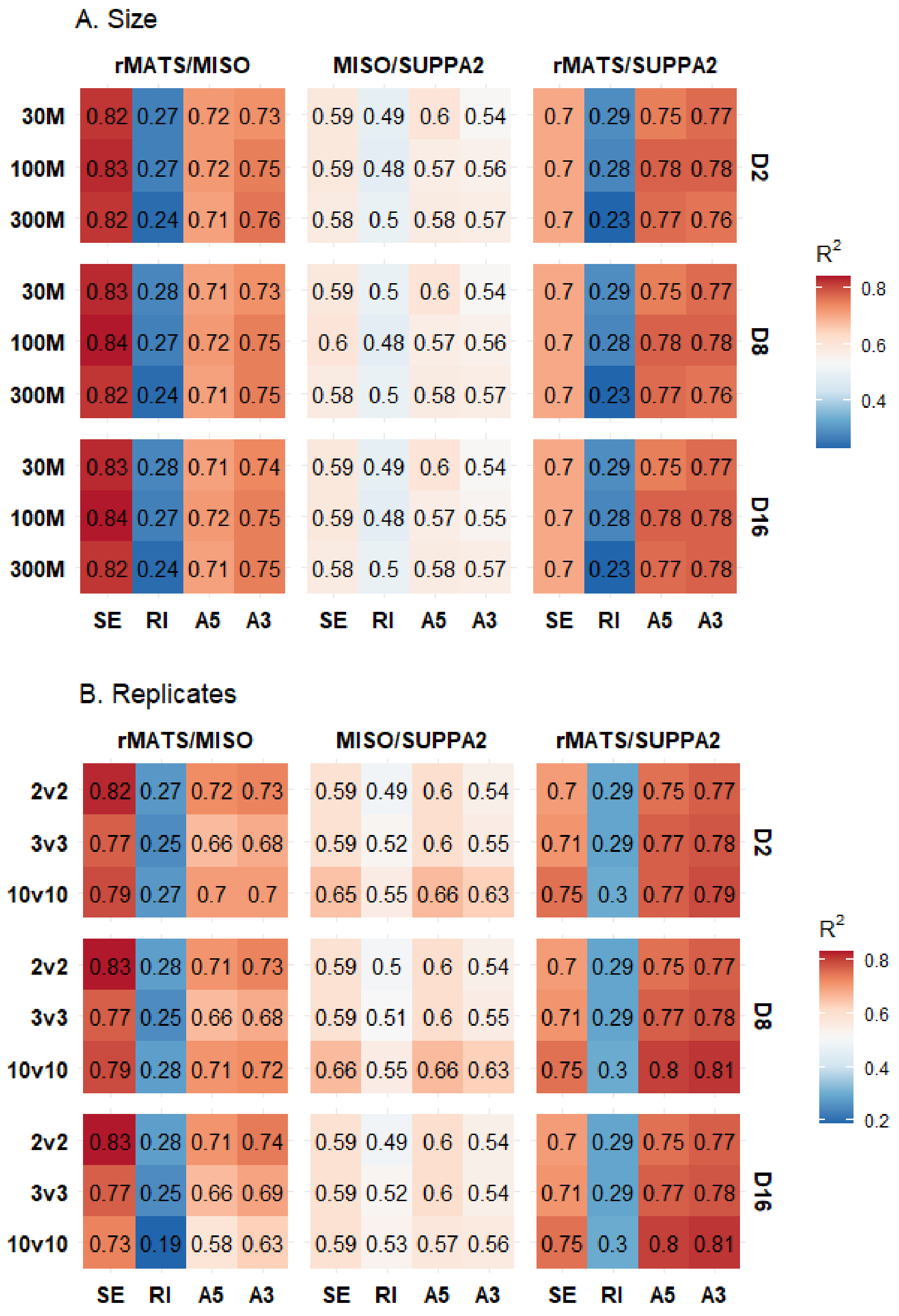

Supplemental Figure 4. Correlation matrix of concordance of all matched events between tools. Linear regression coefficients are shown for each read depth (**A**) or amount of replicates (**B**) for each virtual machine per tool combination (MATS/MISO, MISO/SUPPA2, and MATS/SUPPA2). Event types are: SE, spliced exon; RI, retained intron, A5, alternative 5'splice site; A3, alternative 3' splice site. D2 – D2 virtual machine; D8 – D8 virtual machine; D16 – D16 virtual machine.

Supplemental Figure 5

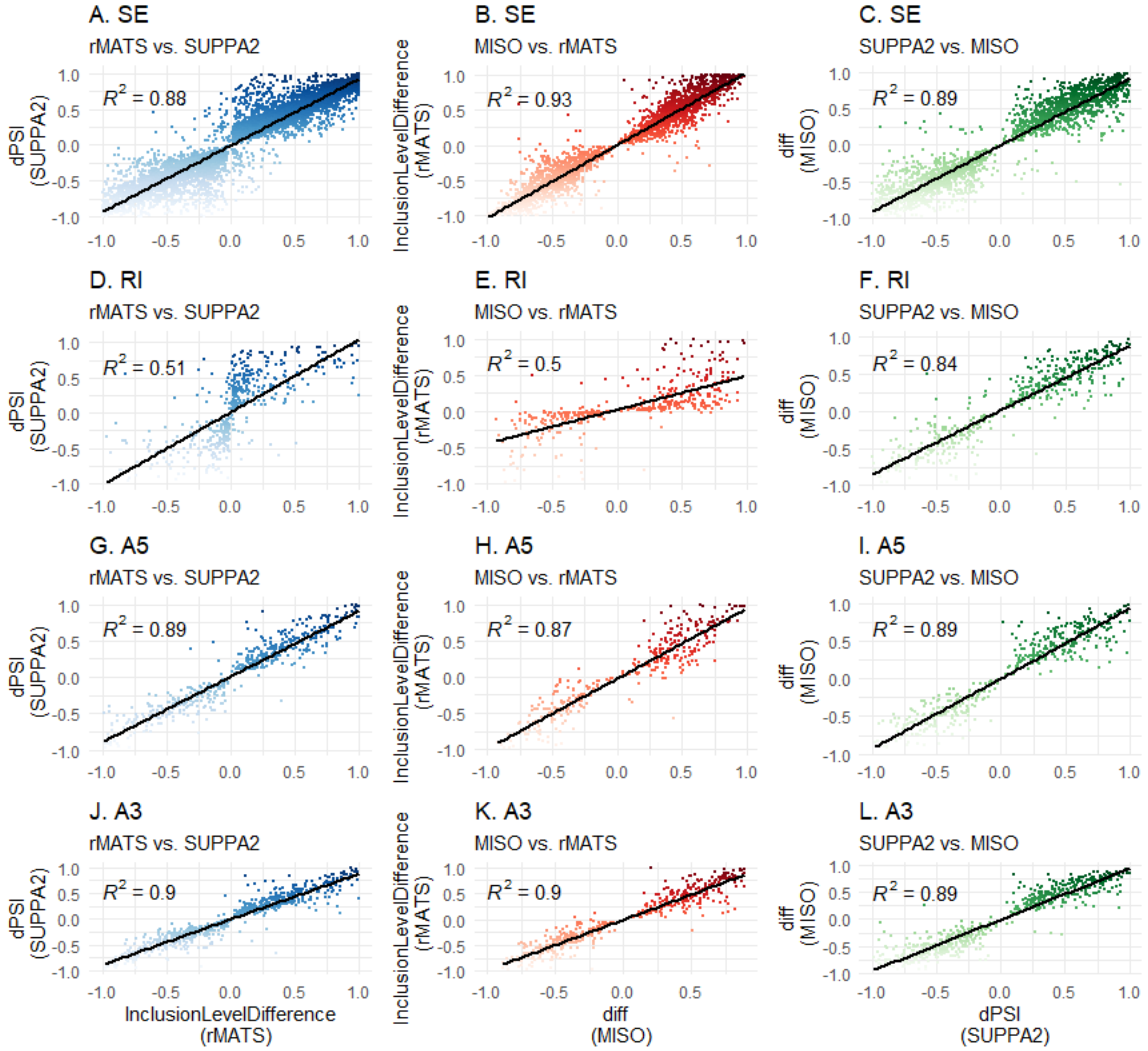

Supplemental Figure 5. Scatterplots of D16 10v10 analyses (significant events) for each event type.

Scatterplots show relative splicing abundance for each tool (rMATS – InclusionLevelDifference; MISO – diff; SUPPA2 – dPSI) and each event type (SE – spliced exon; RI – retained intron; A5 – alternative 5' splice site; A3 – alternative 3' splice site). Regression coefficient is depicted as R^2. Linear regression line is shown in black. 5A-C – SE; 5D-F – RI; 5G-I – A5; 5J-L – A3. Left column – rMATS vs. SUPPA2; middle column – MISO vs. rMATS; right column - SUPPA2 vs. MISO.

Supplemental Figure 6

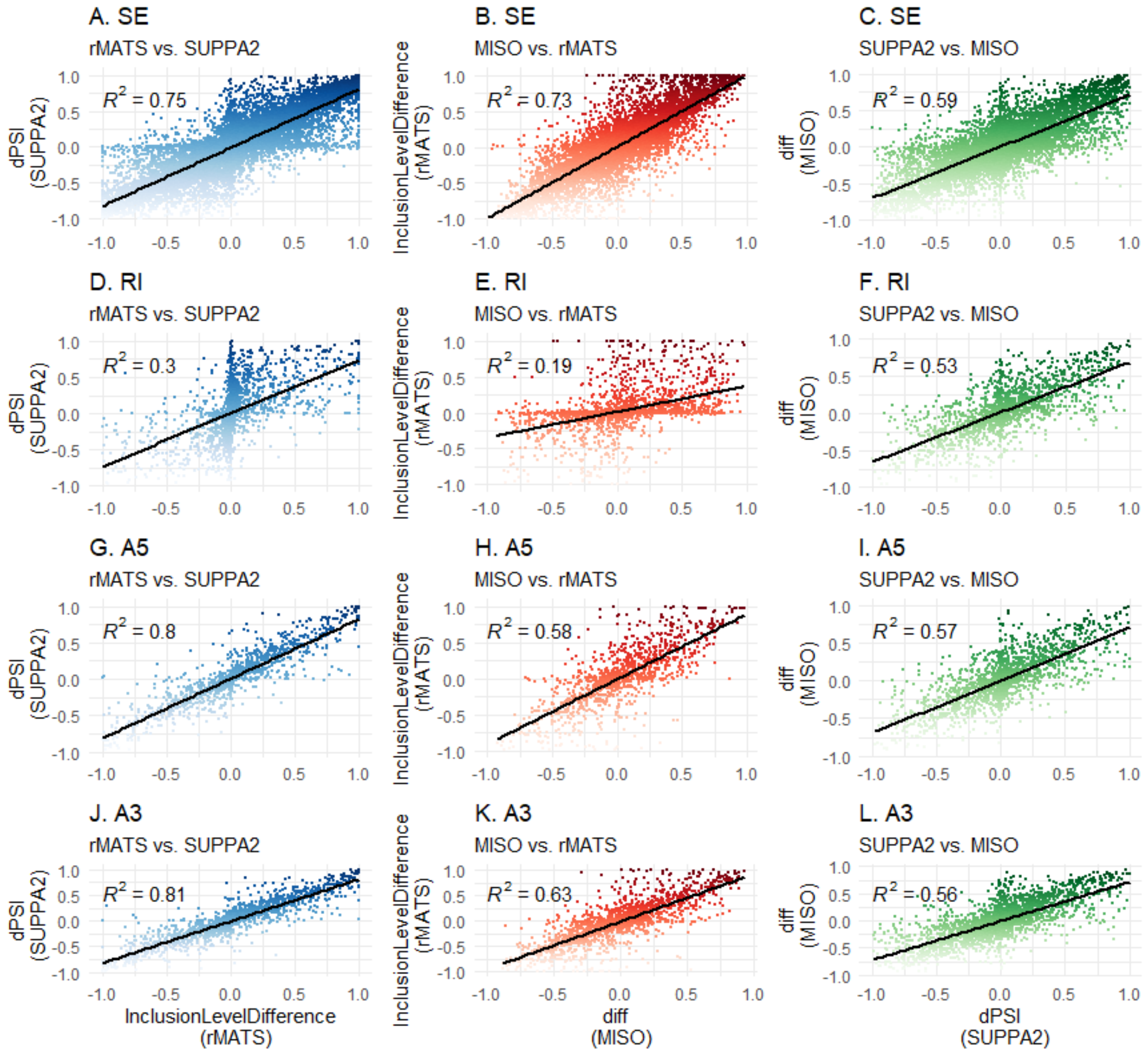

All events D16 10v10.

Supplemental Figure 6. Scatterplots of D16 10v10 analyses.(all events) for each event type.

Scatterplots show relative splicing abundance for each tool (rMATS – InclusionLevelDifference; MISO – diff; SUPPA2 – dPSI) and each event type (SE – spliced exon; RI – retained intron; A5 – alternative 5' splice site; A3 – alternative 3' splice site). Regression coefficient is depicted as R^2. Linear regression line is shown in black. 5A-C – SE; 5D-F – RI; 5G-I – A5; 5J-L – A3. Left column – rMATS vs. SUPPA2; middle column – MISO vs. rMATS; right column - SUPPA2 vs. MISO.
